# Recent Advances in Blood Cell-Inspired and Clot-Targeted Thrombolytic Therapies

**DOI:** 10.1155/2023/6117810

**Published:** 2023-02-17

**Authors:** Anastasia Sheridan, Ashley C. Brown

**Affiliations:** ^1^Joint Department of Biomedical Engineering of University of North Carolina, Chapel Hill and North Carolina State University, Raleigh, NC 27695, USA; ^2^Comparative Medicine Institute, North Carolina State University, Raleigh, NC 27606, USA; ^3^Department of Material Science and Engineering, North Carolina State University, Raleigh, NC 27606, USA

## Abstract

Myocardial infarction, stroke, and pulmonary embolism are all deadly conditions associated with excessive thrombus formation. Standard treatment for these conditions involves systemic delivery of thrombolytic agents to break up clots and restore blood flow; however, this treatment can impact the hemostatic balance in other parts of the vasculature, which can lead to excessive bleeding. To avoid this potential danger, targeted thrombolytic treatments that can successfully target thrombi and release an effective therapeutic load are necessary. Because activated platelets and fibrin make up a large proportion of clots, these two components provide ample opportunities for targeting. This review will highlight potential thrombus targeting mechanisms as well as recent advances in thrombolytic therapies which utilize blood cells and clotting proteins to effectively target and lyse clots.

## 1. Overview of Thrombotic Complications and Treatment Limitations

Excessive thrombus formation can lead to life-threatening conditions such as stroke, myocardial infarction, pulmonary embolism, or disseminated intravascular coagulation. These conditions can be treated surgically to break up thrombi and restore blood flow; however, less invasive methods are preferred to avoid surgical costs and complications such as infection or a reaction to anesthesia. The most common noninvasive method is fibrinolytic or thrombolytic therapy which uses systemic delivery of fibrinolytic agents such as urokinase, tissue plasminogen activator (tPA), streptokinase, reteplase, and tenecteplase [1, 2]. Fibrinolytic agents activate plasminogen into plasmin which cleaves fibrin to break down clots. The main complication of systemic delivery of thrombolytics is excessive bleeding which can result in hematomas at puncture sites, ecchymosis, hemoptysis, or intracranial bleeding [3]. The risk of a major bleeding event varies tremendously based on the fibrinolytic agent and the condition being treated. Daley et al. reviewed the bleeding rates when treating acute pulmonary embolisms with varying fibrinolytic agents in previous studies [4]. They found that systemic treatment can result in bleeding risks in as many as 32.9% of patients. Off-target delivery of thrombolytics also limits the amount that reaches thrombi, thereby increasing the required drug dosage. Furthermore, uptake by the reticuloendothelial system is another large hurdle to drug delivery. Given these issues, nanoparticles that target thrombi and go unrecognized by the reticuloendothelial system are of great interest to treat thrombotic complications. This review highlights recent advancements in thrombolytic therapies that utilize, mimic, and target blood cells and clotting proteins. A summary of technologies is provided in Table 1.

## 2. Mechanisms and Cell Types Contributing to Thrombosis

Maintaining hemostatic balance between procoagulant and anticoagulant factors is imperative so that the body can respond efficiently and effectively to injury. Under steady state conditions, blood fluidity is maintained by antithrombin, proteins *C*, *S*, and *Z*, and a tissue factor pathway inhibitor [5]. After injury, the rate of activation of these proteins is decreased, downregulating their anticoagulant mechanisms. As a result, the conversion of prothrombin into thrombin is upregulated. Thrombin converts fibrinogen into fibrin, creating a fibrin clot, which is then crosslinked by factor XIIIa. The rate of activation of these proteases and complexes contributes to both thrombus formation and maintaining vessel integrity. However, if the rates of activation are not balanced, bleeding or thrombosis can occur. Besides exposure of the vessel wall, thrombosis can be caused by a variety of factors such as blood pooling or atherosclerotic plaque rupture [6]. Similarly, deficiency of key antithrombotic proteins (antithrombin, protein *C*, and protein *S*) or increased levels of prothrombotic proteins (factors VII, XI, IX, VIII, or von Willebrand factor (vWF)) can also cause hypercoagulation [5].

**Figure 1:**
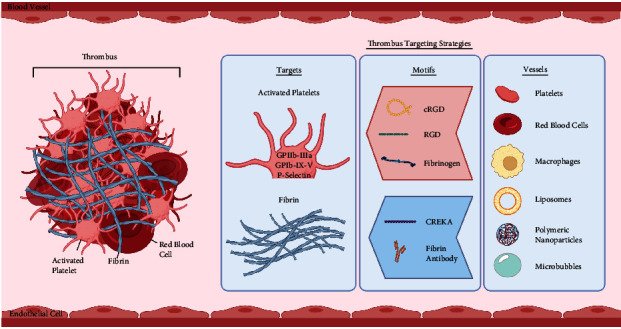
Thrombus targeting strategies for thrombolytic therapies.

Along with coagulation proteins, platelets and other cell types contribute to clot formation and thrombus stability properties. Platelets are anucleated cells with a 2-3 *μ*m diameter, ∼10-day half-life, and a relative abundance of ∼150,000–400,000/*μ*l of blood [7–9]. Platelets play a large role in primary hemostasis; once bound to vWF, exposed collagen, or soluble platelet agonists, platelets change shape, accumulate and aggregate at injury sites, and interact with fibrin(ogen) to form a plug to cease blood flow from a damaged vessel [10–13]. Since platelets comprise a large portion of thrombi, they are excellent targets for thrombolytic therapies. One possible platelet target is P-selectin (13,000/platelet). This glycoprotein exists in *α*-granules until platelet activation, where it is translocated to the membrane. Glycoprotein (GP) Ib-IX-V complex (50,000/platelet) is another potential target as it is the second most common receptor on platelets; however, the most abundant and most commonly targeted is *α*_IIb_*β*_3_, also called the GPIIb-IIIa complex [8, 13–15]. These receptors are located on platelet membranes (80,000–100,000/platelet) and intracellularly within granules (20,000–40,000/platelet). Once platelets become activated by initial agonist binding, these receptors undergo conformational changes to display a high affinity binding site for fibrinogen. Additionally, intracellular receptors translocate to the plasma membrane, and there is evidence suggesting that they are already complexed with fibrinogen to enhance fibrin clot formation [16]. Given the abundance of these receptors on activated platelets, targeting them could be the key to efficient and precise thrombolytic delivery. Common thrombi-specific receptors and platelet- and fibrin-based targeting agents for thrombolytic therapies are illustrated in Figure 1.

While red blood cells (RBCs) are not necessarily major contributors to thrombus formation; some studies indicate that elevated RBC levels could lead to serious thrombotic conditions such as cardiovascular disease or venous thromboembolism [17–19]. A recent review summarizes evidence supporting RBCs' influence on both hemostasis and thrombosis [20]. Some proposed mechanisms of how RBCs encourage thrombosis include contributing to platelet margination toward the vessel wall, therefore increasing deposition of platelets on thrombi [21–23]. Also, when RBCs are present in clots, they reduce the degree of clot contraction, which is an imperative step to promote blood flow past large thrombi [24, 25]. Despite these thrombotic associations, RBCs also provide advantageous characteristics to aid in therapeutic delivery to thrombus sites. These anuclear cells are biocompatible, deformable, and abundant, having a concentration of ∼4.2 − 6.1 × 10^9^/mL of blood. RBCs have a large surface area of ∼140 um^2^ [18, 26] and a long natural half-life of ∼70–120 days [27–29]. While RBCs do not have direct thrombus targeting abilities intrinsically, they can be engineered with motifs or nanoparticles to create a camouflaged and targeted therapeutic vehicle.

White blood cells (WBCs) play an important role in the immune system and thrombosis [30]. In an inactivated state, leukocytes release anticoagulant factors such as endothelial protein C receptors (EPCR) and tissue factor (TF) pathway inhibitors. Once activated during inflammation, they release procoagulant factors including TF and matrix metalloproteinases [31, 32]. However, activated leukocytes have also been shown to release microparticles which contain both procoagulant TFs and anticoagulant factors such as EPCR [33]. Additionally, leukocytes form complexes with activated platelets via P-selectin, contributing further to aggregation [34]. Given that activated WBCs promote thrombosis in multiple ways, few researchers have used WBCs to treat thrombotic conditions. However, Burnouf et al. created a proof-of-concept for macrophage-loaded thrombolytic therapy by loading macrophages with polypyrrole-polyethylenimine nanocomplexes which target thrombi and lyse clots via near infrared (NIR) irradiation [35]. They saw successful clot ablation of NIR treatments combined with test nanoformulations in a rat femoral vascular thrombosis model; however, they did not test the full macrophage-based nanoformulation in vivo. Another group combined neutrophils with urease catalysis micromotors to create a targeted treatment that can overcome blood flow resistance [36]. The micromotors produce ammonia and carbon dioxide to propel urokinase-silver nanoparticle-loaded neutrophils. Once at the thrombus site, the neutrophil will expel the internal cell contents to form neutrophil extracellular traps. These altered neutrophils were tested in vivo in carotid thrombosis and lower extremity arterial thrombosis mouse models. They demonstrated excellent thrombus targeting times (<30 minutes) and rapid thrombolysis in both models. These two studies are pioneering examples of how WBCs can be used to treat thrombotic complications.

## 3. Platelet-Derived, Inspired, and Targeted Thrombolytic Therapies

**Figure 2:**
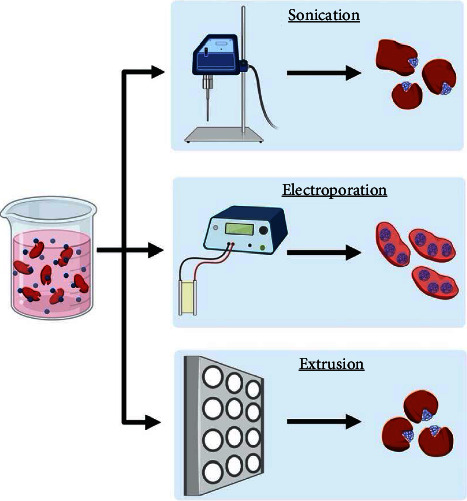
Platelet and thrombolytic therapeutic fusion techniques. Platelets can be loaded with thrombolytics through sonication, electroporation, or extrusion. Sonication uses ultrasonic waves to fuse cargo and membranes together [44], while electroporation exposes membranes to electrical waves, creating pores through which cargo can enter [45]. Extrusion requires both cargo and membranes to be run through a porous membrane, where mechanical pressure forces them to interact [46].

**Figure 3:**
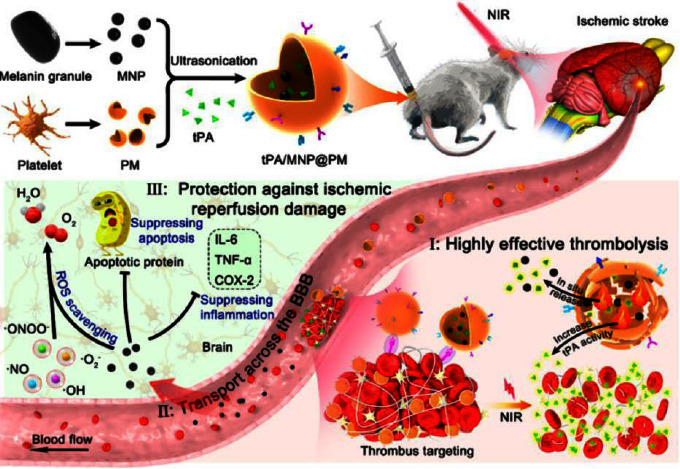
Schematic illustration of the fabrication of tPA and melanin nanoparticle-loaded platelets and their application for cascaded thrombolysis and neuroprotection mechanisms in ischemic stroke. Figure 3 is reproduced from Yu et al. [43], with permission from Elsevier.

Platelets play an imperative role in hemostasis and thrombosis, and while platelets are primarily thought of as contributing to thrombotic outcomes, researchers have found ways to incorporate platelets into thrombolytic therapies to treat stroke, reperfusion injuries, pulmonary embolism, and other thrombotic conditions. Besides thrombus targeting, most of these complex therapeutics use the platelet membrane as a “cloak” to aid in immune system evasion through CD47 expression. Additionally, CD55 and CD59 expression prevent complement activation [37]. Therefore, platelet-cloaks extend circulation time and half-life of the therapeutic in addition to limiting off-target effects which can be very costly when delivering anticoagulant, antiplatelet, or fibrinolytic agents [38]. The main two phases to create cell-membrane camouflaged nanoparticles are (1) platelet membrane isolation and (2) therapeutic loading [39, 40]. For membrane isolation, platelets are carefully lysed via freeze-thaw cycles [41–43] and centrifuged to separate membranes from internal cell contents. Biological or chemical cargo is then loaded through sonication, electroporation, or extrusion [40] (Figure 2). Researchers must determine which method is most appropriate for their specific therapeutic and application. Recently, there have been exciting complex cargo designs that go beyond simple drug delivery and expand the potential impact of platelet-derived therapeutics. Li et al. designed a nanocarrier that utilized both the natural targeting ability of platelets and a magnetic field to localize particles to stroke lesions [41]. Their nanocarriers, platelet membrane envelopes loaded with *γ*-Fe_2_O_3_ and L-arginine magnetic nanoparticles (PAMNs), use a magnetic field to target thrombus sites. There, L-arginine stimulates nitric oxide (NO) release from endothelial cells, inducing vasodilation and helping restore blood flow to the tissue. In a mouse model of ischemia, PAMNs successfully localized to stroke lesions, released NO, and aided in blood vessel expansion, therefore reducing platelet aggregation and restoring blood flow to the local tissue. Yu et al. also developed a therapeutic that utilizes an external stimulus, but for the purpose of thrombolysis as opposed to targeting [43]. They loaded platelet membranes with tPA to promote fibrin lysis and melanin nanoparticles to scavenge free radicals. The particles assisted with both thrombolysis and neuroprotection from reperfusion injury. Their proposed mechanism utilized platelets' natural targeting ability to reach thrombus sites where NIR irradiation would be administered. The irradiation photoconverts melanin, causing platelet membrane rupture to release tPA and stimulate fibrinolysis. Meanwhile, melanin nanoparticles would cross the blood brain barrier, scavenge free radicals, and suppress inflammation to reduce the risk of ischemia-reperfusion injury (Figure 3). In a rodent cerebral thrombosis model, groups that received therapeutic-loaded platelets showed a significant decrease in the infarct size, lower reactive oxygen species presence, and greater blood stream recovery. Through the examples mentioned, there are obvious benefits for using platelet-derived therapeutics such as improved thrombus targeting abilities, immune evasion, and elongated half-lives [38].

While the use of platelet-derived therapies clearly has promise for treating thrombotic complications, there are some major hurdles that need to be overcome regarding large-scale production of platelet-derived therapies [47]. Currently, there are no established protocols to scale up production of these products. In addition, these strategies all require the use of natural platelets, which relies on donor availability. There is a current blood shortage that will limit platelets available for production. Finally, the use of real platelets limits the product's shelf life. Given these challenges, research focused on large-scale production of platelet-derived therapeutics should be a priority.

Some researchers, including our own group, are avoiding these hurdles by opting to mimic platelets instead [48, 49]. Our lab's approach to creating synthetic platelets employs a micron-sized hydrogel, or microgel, coupled to a fibrin-binding motif [50]. The microgel is ultra-low crosslinked (ULC) poly (N-isopropylacrylamide) copolymerized with acrylic acid (AAc). Similar to native platelets, these particles are ∼1 *μ*m in diameter in solution, and their high deformability allows them to mimic activated platelet morphology [51]. Our synthetic platelets, termed platelet-like-particles (PLPs), are created by conjugating ULC microgels to fibrin-specific motifs; we have explored several different fibrin-binding elements including nanobodies, full-length antibodies, and peptides. The fibrin-binding ability allows PLP to bind to fibrin at injury sites. We have shown in rodent trauma models that following intravenous injection, PLPs target wound sites and interface with nascent fibrin fibers to augment clotting and decrease bleeding in vivo [50, 52]. We have also demonstrated that PLPs mimic platelet-mediated clot retraction, a function which stabilizes clots and promotes long term healing responses. This behavior is due to a Brownian Wrench type mechanism which arises from the combination of fibrin binding with the highly deformable microgel. In addition to their usefulness in treating bleeding after trauma, PLPs have also shown utility in treating chronic wounds [51], mitigating neuroinflammation after traumatic brain injury [53], and fighting infection when loaded with gold and silver nanoparticles [54, 55]. The ability to target sites of fibrin also provides the ability to target existing thrombi to deliver fibrinolytic drugs. Our group has explored this approach and found that more highly crosslinked nanogels are more ideal for drug delivery. These fibrin-targeted nanogels are described in more detail in the fibrin-targeting section below.

**Figure 4:**
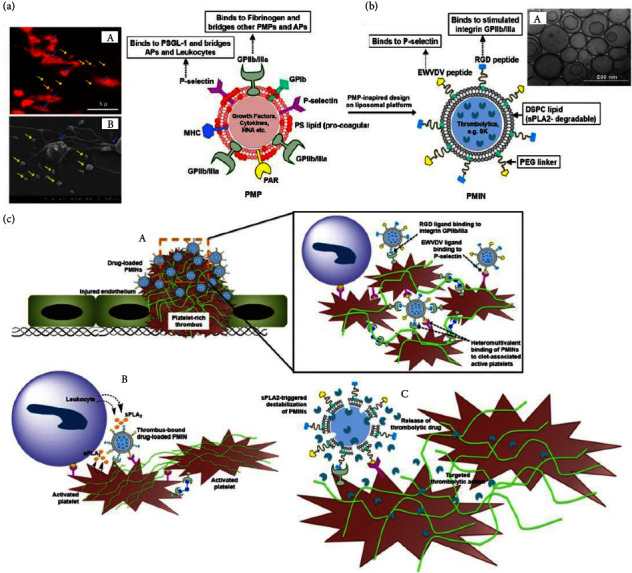
Schematic representation of platelet-derived microparticles (PMP) (a) showing characteristic surface entities, with (A) showing a representative red fluorescence image of PE-anti-CD62P stained active platelets (stained for P-selectin and shown with blue arrows) shedding PMPs (shown with yellow arrows), and (B) showing a representative high resolution SEM image of active platelet shedding microparticle, with PMPs (shown with yellow arrows) visible as submicron vesicular structures; (b) schematic representation of PMP-inspired nanovesicle (PMIN), with (A) showing a representative cryo-TEM image of PMINs developed for the studies; (c) the envisioned mechanism of targeted thrombolytic action using PMINs, where (A) PMINs can actively anchor onto platelet-rich thrombi by virtue of heteromultivalent binding to integrin GPIIb-IIIa and P-selectin on active platelets, (B) clot-bound PMINs get acted upon by sPLA2 enzymes secreted from leukocytes and active platelets in the thrombus milieu, and (C) drug released from degraded PMINs renders site-specific fibrinolysis. Figure 4 is reproduced from Pawlowski et al. [48], with permission from Elsevier.

Other researchers have also mimicked platelets for hemostatic applications and targeted delivery of fibrinolytics. Pawlowski et al. took inspiration from a previously developed platelet-derived microparticle nicknamed “platelet dust” [48]. Platelet dust was more formally called platelet-derived microparticles (PMPs) and was found to play important roles in cellular signaling, coagulation, and homeostasis [56, 57]. Pawlowski et al. created PMP-inspired nanovesicles (PMINs) that utilize a liposomal platform that targets activated platelets through GPIIb/IIIa and P-selectin-specific RGD and EWVDV peptides. Once the PMINs accumulate at thrombi, leukocyte-driven enzymatic degradation causes a breach in the liposomal membrane, thereby releasing thrombolytic cargo streptokinase (Figure 4). In a carotid artery thrombosis mouse model, PMINs effectively targeted thrombi and delayed vessel occlusion while not impacting quiescent platelets and systemic hemostatic capabilities. Given these promising results, this platelet-mimetic platform could be used to deliver antiplatelet, anticoagulant, and fibrinolytic agents to treat a variety of thrombotic conditions.

## 4. Red Blood Cell-Derived and Inspired Thrombolytic Therapies

**Figure 5:**
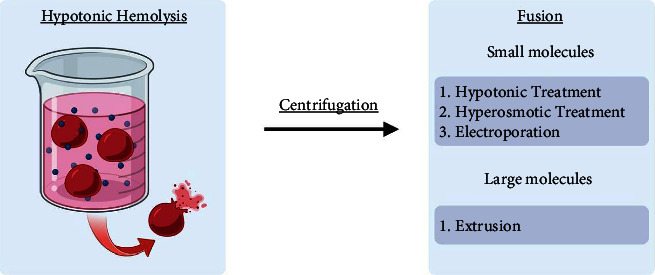
RBC lysis and fusion methods. Schematic illustrating RBC lysis, centrifugation, and fusion for small and large molecules.

Given RBCs' abundance, larger size, and lengthened life span, RBCs provide another ingenious platform to camouflage and improve the delivery of thrombolytic therapies. Like platelets, RBC membranes are used as a cloak for camouflage. For this purpose, RBCs must first be lysed and separated from internal contents. One popular method of RBC lysis is hypotonic hemolysis [58–60] which involves exposing RBCs to a hypotonic buffer where they begin to swell until rupture/lysis, followed by centrifugation to separate internal contents and membranes. Drugs or nanoparticles can then be encapsulated by or conjugated onto those RBC membranes. Guido et al. summarize various techniques for encapsulation and conjugation of drugs into/onto RBCs [27]. For encapsulation, there are three main methods: exposure to the hypotonic environment, exposure to the hyperosmotic environment, and electroporation (Figure 5). All methods involve temporarily opening pores to diffuse drugs into the RBCs. Extrusion has also been used for the fusion process due to the larger size and complexity of the cargo [59, 60].

**Figure 6:**
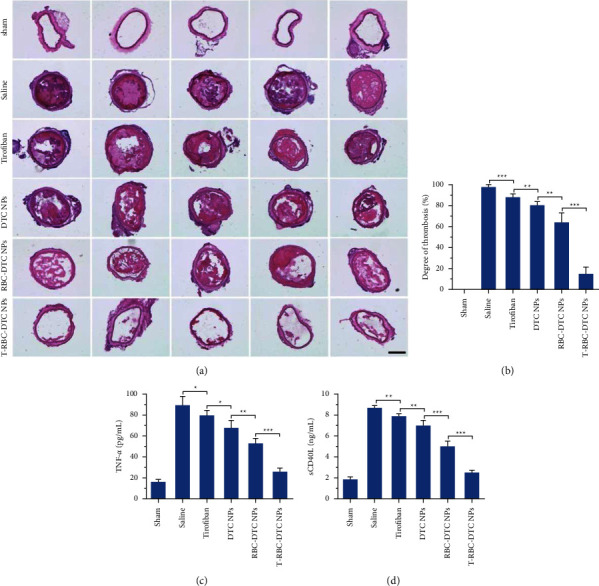
Therapeutic potential of the T-RBC-DTC NPs in the FeCl_3_-induced carotid artery thrombosis mouse model. (a) *H* & *E* staining of the carotid arteries from the mice subjected to various treatments. Scale bar: 200 *μ*m. (b) Quantitative analysis of the thrombosis degree. Data are shown as mean ± SD (*n* = 5). (c) The levels of TNF-*α* after various treatments as detected by ELISA. Data are shown as mean ± SD (*n* = 5). (d) The levels of sCD40 L after various treatments. Data are shown as mean ± SD (*n* = 5). ^*∗*^*p* < 0.05, ^*∗∗*^*p* < 0.01, ^*∗∗∗*^*p* < 0.001. Figure 6 is reproduced from Zhao et al. [60], with permission from Elsevier.

For conjugation, therapeutic molecules can be attached to RBC membranes through external linkers such as streptavidin [61, 62] or RBC-specific antibodies [63]. Since both encapsulation and conjugation require RBC manipulation, hemolysis, loss of glycocalyx or protection of surface conjugated ligands, and change in osmotic pressure can occur [27]. It has also been shown that conjugation with rigid nanoparticles (polystyrene), as opposed to soft nanogels (lysozyme-dextran), induces RBC agglutination and increases RBC stiffness which can impact its ability to maneuver microvasculature and penetrate clots [64]. Careful consideration must be made when deciding how to incorporate thrombolytic elements into RBC membranes. Historically, RBC membranes started off as camouflage for basic anticoagulants or fibrinolytics such as heparin [65] and tPA [61, 66]. More advanced examples have recently been described. For example, Shao et al. created an erythrocyte membrane-cloaked Janus polymeric motor (JPM) nanoparticle (EM-JPM) composed of heparin and chitosan multilayers that were partially gold coated [58]. EM-JPMs combine mechanical force, heat, and anticoagulant abilities to break up thrombi. They showed promising targeted and fibrinolytic abilities in an in vitro fibrin clot model under both static and flow conditions; however, animal studies are necessary for further validation. To address multiple issues associated with thrombotic disorders, Zhao et al. designed a fibrin-targeted RBC membrane-cloaked dextran-tirofiban conjugate (T-RBC-DTC) nanoparticle that has enhanced targeting ability, stimulus-controlled drug delivery, long circulation time, and ROS scavenging capabilities [60]. T-RBC-DTC nanoparticles are cloaked in an RBC membrane and conjugated to the fibrin-targeting peptide, Cys-Arg-Glu-Lys-Ala (CREKA). The antithrombotic cargo, dextran-tirofiban conjugate nanoparticles, utilizes dextran as an H_2_O_2_ responsive linker that can be oxidized by ROS. This responsive linker both scavenges ROS and releases tirofiban, an antithrombotic agent. In a carotid thrombosis mouse model, they saw sufficient accumulation at thrombus sites and enhanced antithrombotic activity compared to free tirofiban which can be seen in Figure 6. The researchers noted when designing this therapeutic that they chose an RBC coating over a platelet coating because the antiplatelet agent, tirofiban, could compromise the targeting ability of the platelets. Interestingly, another researcher was also considering both platelet and RBC coatings and decided to put the two cells up against each other [67]. Their results indicated that while both RBC (RFNP) and platelet (PFNP) membrane-coated fullerenol-loaded MSNs enhanced circulation times, reduced macrophage phagocytosis, showed excellent blood compatibility, biosafety, and reduced bleeding times; only RFNPs significantly enhanced thrombolysis in vivo when compared to the free drug. Chen et al. predicted that when the platelets were collected, they were inactivated by reagents such as ethylenediaminetetraacetic acid which most likely reduced their fibrin-binding affinity. Overall, benefits of using RBCs over platelets are their abundance, larger size, and ability to be loaded with antiplatelet agents without compromising membrane receptors.

**Figure 7:**
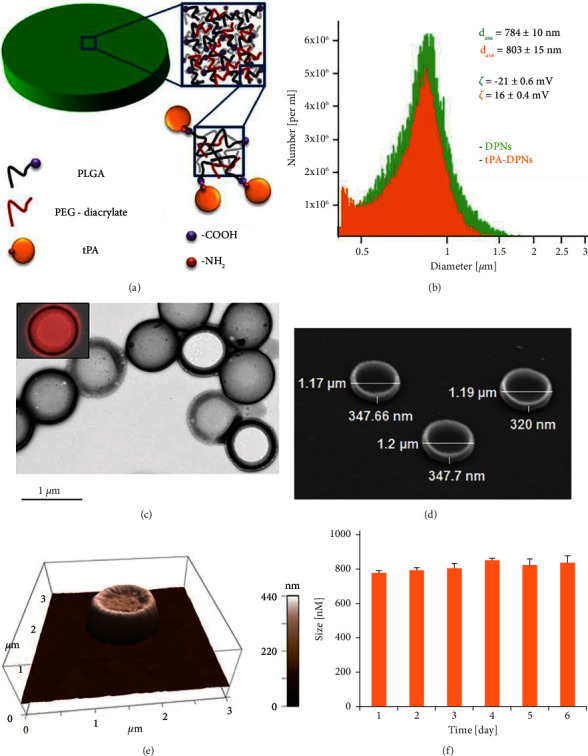
Physicochemical properties of discoidal polymeric nanoconstructs associated with tPA molecules (tPA-DPNs). (a) Schematic representation of tPA-DPNs, highlighting the porous structure of DPNs and their direct conjugation with tPA. (b) Multisizer analysis of DPNs (green) and tPA-DPNs (orange). (c) The TEM image of DPNs demonstrating the circular shape with a base diameter of ∼1000 nm. The upper-left inset shows a fluorescent microscopy image of a RhB-DPN superimposed on its TEM image. (d) The SEM image of DPNs demonstrating the diameter of ∼1200 nm and the height of ∼347 nm. (e) The AFM image of DPNs demonstrating the diameter of ∼1100 nm and the height of ∼300 nm. (f) Size stability of tPA-DPNs in PBS at 37°C via DLS analysis (*n* = 3). Figure 7 is reproduced from Colasuonno et al. [68].

While RBC-derived nanoparticles have shown great promise for delivery of antithrombotic therapies, they also share similar limitations with platelets including issues related to scaling up and blood product shortages. One major limitation that is unique to RBCs is blood incompatibility which can cause serious issues such as hemolysis and agglutination [27]. Using RBCs that share the same blood type as the patient is ideal; however, that is not always possible. To avoid all issues associated with using real RBCs, some researchers have found ways to mimic RBCs. Colasuonno et al. designed a porous hydrogel-based particle that mimics the discoidal shape of RBCs [68]. They directly conjugated tPA to PLGA and polyethylene glycol (PEG) matrices via activated carboxylic groups (tPA-DPNs) and used silica molds to achieve a discoidal shape. Refer to Figure 7 for a schematic and characterization. In a thrombosis mouse model, tPA-DPNs dissolved almost 90% of blood clots, while free tPA only dissolved 40%, verifying the importance of targeted delivery.

## 5. Fibrin-Derived, Inspired, and Targeted Thrombolytic Therapies

Fibrin and its precursor, fibrinogen, are common components in thrombolytic therapy mechanisms due to fibrin's abundance in clots and fibrinogen's binding motifs. Fibrinogen contains RGD peptides which target and bind to activated platelets via integrin *α*_II*b*_*β*_3_ [69]. Normally, this leads to further platelet accumulation; however, researchers have utilized this fibrinogen-derived targeting motif to break up clots instead of enhancing them. Chung et al. used a fibrinogen-derived RGD peptide (Gly-Arg-Gly-Asp) to hone their tPA-loaded PLGA nanoparticles to thrombus sites in a clot-occluded tube model [70]. While their tPA-loaded PLGA nanoparticles did not have the shortest clot lysis time when compared to their nontargeted nanoparticles, they did have the highest percentage of digested clots. Similarly, Absar et al. also used a fibrinogen-derived *γ*-chain *C*-terminal peptide (CQQHHLGGAKQAGDV) to target their albumin-camouflaged tPA-loaded nanoparticles to thrombi [71]. Their results were also not ideal because nanoparticle thrombolytic activity was similar to free tPA. To improve upon these therapies, Ye et al. incorporated entire fibrinogen molecules onto the dopamine core of their nattokinase-loaded microcapsules [49]. These microcapsules showed excellent antithrombotic performance in vitro through sharp dissolution of fibrin clots and a high blood clotting index. While fibrinogen and fibrinogen-derived RGD binding motifs are still commonly used in targeted thrombolytic therapies, cyclic RGD (cRGD) peptides have also grown in popularity. cRGD peptides mimic fibrinogen-derived peptides but have a different conformation that gives them an advantage over linear RGD peptides in regards to specificity and affinity [72]. Huang et al. tested cRGD-conjugated liposomes and linear RGD-conjugated liposomes in a rat carotid injury model and found that cRGD-bound liposomes were significantly better at binding activated platelets [69]. Following this finding, Zhang et al. loaded cRGD-bound liposomes with urokinase and tested them in a mouse mesenteric thrombosis model where they found that liposomal carriers reduced the dosage of urokinase necessary to achieve sufficient thrombolysis by 75% [73]. Similarly, Huang et al. conjugated cRGD to a tPA-loaded lipid nanoparticle, creating a multiarmed nanovesicle which was tested under both static and flow conditions in vitro where the nanoparticles showed efficient tPA release at thrombus sites and clot lysis times similar to free tPA [74]. Further in vivo research will need to be conducted to expand the impact of both fibrinogen-derived and fibrinogen-mimicked thrombolytic therapies. Comparatively, fibrin-targeted thrombolytic therapies have been extensively researched, and more recent advances will be highlighted here. Adzerikho et al. set out to create a thrombolytic nanovesicle that could accomplish both rapid and prolonged fibrinolytic effects [75]. Their liposomal platform incorporated both bound and free streptokinase and was conjugated to FnI-3C, a fibrin-specific antibody. They found that a 40 : 60 ratio of bound to free streptokinase resulted in the largest thrombolytic effect which was 13 times greater than the free drug in a rat thrombosis model.

**Figure 8:**
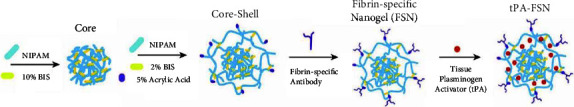
Fibrin-specific nanogel synthesis schematic and characterization. Schematic of core-shell particle synthesis and FSN fabrication followed by subsequent tPA loading through a rehydration technique. Figure 8 is reproduced from Mihalko et al. [77], with permission from Elsevier.

Utilizing a different platform and a fibrin-specific antibody, our lab has developed a fibrin-specific nanogel (FSN) that can be loaded with fibrinolytic agents to treat multiple thrombotic complications such as myocardial infarction (MI) or disseminated intravascular coagulation (DIC) [76–78]. FSNs have a core-shell conformation and are synthesized through polymerization reactions between poly (N-isopropylacrylamide) (pNIPAM) and N,N′-Methylenebis (acrylamide) (BIS), conjugated to an antifibrin fragment *E* antibody, and loaded with the appropriate agent. FSN synthesis can be seen in Figure 8. To treat thrombotic complications, FSNs were loaded with tPA. It was hypothesized that FSNs could prevent premature release of tPA and directly deliver tPA to microthrombi and break them up. In the context of treating thrombotic occlusion associated with MI, tPA-FSNs dual loaded with a small molecule Rho-kinase inhibitor, Y-27632, were found to localize to injured heart tissue and significantly improve left ventricular ejection fraction 2 and 4 weeks post-MI [76]. These studies also showed a significant decrease in the infarct size and signs of fibrosis 4 weeks post-MI. Our group has also used tPA-FSNs to treat the complex thrombotic disorder, DIC. DIC results are due to excessive thrombin generation secondary to many conditions including sepsis, trauma, and pregnancy. Thrombi form throughout the microvasculature and can lead to clotting factor consumption, hemorrhage, multi-organ failure, and, in up to 70% of cases, death. In a rat model of LPS-induced DIC, we found that tPA-FSNs decreased the amount of visible multiorgan microthrombi and increased platelet and D-dimer concentration, both signs of thrombus ablation [77]. Additionally, tPA-FSNs were well tolerated in vivo even up to 20 times the optimized therapeutic dose. In vivo studies also showed tPA-FSNs were cleared within 24 hours [78].

Taking a less biomaterial-centered approach, Li. et al. created a fibrin-targeted treatment with CREKA-modified microbubbles [79]. Under physiologically relevant flow, the CREKA-modified microbubbles bound firmly to thrombi. Zhao et al. also used CREKA as the targeting agent for their therapeutic, but their design used photothermal therapy in addition to thrombolytic therapy to create a nanoparticle that deeply penetrates thrombus sites and ablates them [80]. Their design is a coassembly of a photothermal probe, 1,1′-dioctadecyl-3,3,3′,3′-tetramethylindotricarbocyanine iodide (DiR) and ticagrelor, an antiplatelet drug. This coassembly core is conjugated to CREKA via PEG. In vivo, these nanoparticles target and accumulate in fibrin-rich clots where they are exposed to irradiation. Photoconversion of DiR increases the local temperature which helps break up noncovalent interactions, allowing the ticagrelor to further penetrate clots. This photothermal-amplified antithrombotic effect was tested in a rat carotid arterial thrombosis model. The nanoparticles combined with photothermal therapy outperformed all single therapies and had a thrombolysis therapeutic rate greater than 75%. Fibrin-targeted and fibrinogen-derived therapies, whether alone or combined with other therapeutic methods, have shown great promise for safely delivering thrombolytics and effectively lysing clots.

## 6. Conclusions

For a thrombolytic therapy to be successful, three main things must occur: precise thrombus targeting, efficient load deposition, and effective clot lysis. Most thrombi are composed mainly of activated platelets and fibrin, making these the two main targets. Most of the technologies mentioned in this review target only one of those components; however, targeting both could be a potential avenue for thrombolytic therapies. In fact, this combined targeting technique has been shown to anchor clots more efficiently with a lower ligand density than single-targeted nanoparticles [81]. Another targeting aspect to consider is the use of external guidance. While thrombolytic therapies that utilize platelets and RBCs as camouflage do aid in targeting, only platelets have proven clot-targeting mechanisms. External guidance, such as magnets or NIR, is sometimes necessary to direct the therapeutics to clots. Similarly, most of the technologies mentioned in this review utilize diffusion for drug release, but some also use NIR to release the thrombolytic cargo. These combined therapies may have enhanced clot targeting and clot ablating capabilities, but their translatability is hindered due to the sophisticated machinery necessary for the therapeutic to be effective.

While RBCs do not have an internal targeting mechanism, RBCs may be better at loading vesicles because they are not limited by the chemical nature of the cargo as opposed to platelets. Similarly, blood-cell mimics provide the same advantageous characteristics of blood-cell-derived drug delivery systems but have the additional benefit of not relying on donor availability or being concerned with dangerous consequences of blood incompatibility. Still, the immunogenicity of certain artificial materials should be carefully reviewed. Multiple technologies discussed in this review used PEG as a linker or a nanoparticle component which could create challenges later when assessing immune responses in humans. The physiomechanical properties of these therapeutics are also important to consider. The particle size, shape, and stiffness affect intravascular drug delivery systems [82]. While most drug delivery systems are spherical due to energy minimization, anisotropic shapes have demonstrated better vessel wall margination and adhesion in a hemodynamic environment. Similarly, stiff microsized nanoparticles marginate towards vessel walls more effectively, but soft and flexible nanosized particles navigate microvasculature more efficiently. The geometry and material properties of these drug delivery systems must be carefully tuned to promote thrombus targeting.

The application of these therapeutics to both arterial and venous clots should also be examined. Most therapeutics were tested in arterial clots which are mainly composed of platelets and fibrin, while venous clots are composed mainly of RBCs and fibrin [83]. For this reason, fibrin-targeted and derived thrombolytics may be preferred because they can target both arterial and venous clots. Overall, blood-cell inspired and clot-targeted therapies have the potential to make delivering thrombolytic agents safer and more effective to improve the outcomes of patients suffering from life-threating thrombotic disorders. Additionally, clot-targeted fibrinolytic therapies could improve therapy for recalcitrant clots by enhancing the accumulation/concentration of fibrinolytics directly at the clot.

## Figures and Tables

**Table 1:** Blood cell-inspired and clot-targeted thrombolytic therapy technologies, targeting agents, and animal models in which they were tested.

Blood cell/protein involvement	Technology	Targeting mechanism	Animal models	Source
White blood cell-based	Macrophage-based polypyrrole-polyethylenimine nanocomplex	Natural thrombus targeting	Rat femoral vascular thrombosis model	[35]

Platelet-based and targeted	*γ*-Fe_2_O_3_ and L-arginine magnetic nanoparticle-loaded platelets	Natural thrombus targeting, magnetic field	Mouse focal cerebral ischemia model	[41]
	tPA and melanin nanoparticle-loaded platelets	Natural thrombus targeting	Mouse/Rat cerebral thrombosis model and MCAO model	[43]
	tPA-conjugated and PLGA-loaded platelets	Natural thrombus targeting	Mouse pulmonary embolism model, mesenteric artery thrombosis model, and MCAO model	[42]
	Fullerenol mesoporous silica nanoparticle-loaded platelets	Natural thrombus targeting	Rat carotid thrombosis model	[67]

Platelet-inspired and targeted	Streptokinase-loadedplatelet-derived microparticle-inspired nanovesicles	RGD and EWVDV (CDAEWVDVS) peptides	Mouse carotid arterial thrombosis model	[48]

RBC-based	Heparin-loaded erythrocytes	None reported	Dog biocompatibility model	[65]
	tPA-loaded RBCs	None reported	Pig cerebral hypoxia/ischemia model	[61]
	tPA-loaded RBCs	None reported	Mouse carotid arterial thrombosis model and pulmonary embolism model	[66]
	Erythrocyte membrane-cloaked janus polymeric motor nanoparticles	NIR	None reported	[58]
	Fullerenol mesoporous silica nanoparticle-loaded RBCs	None reported	Rat carotid arterial thrombosis model	[67]

RBC-based and fibrin-targeted	Fibrin-targeted RBC membrane-cloaked dextran-tirofiban conjugate nanoparticle	CREKA	Mouse carotid arterial thrombosis model	[60]

RBC-inspired	tPA-conjugated PLGA/PEG nanoparticles	None reported	Mouse thrombosis model	[68]

Fibrinogen-derived and platelet-targeted	tPA-loaded PLGA nanoparticles	RGD peptides	None reported	[70]
	Thrombin-cleavable camouflaged-tPA	Fibrinogen g-chain *C*-terminal peptide (CQQHHLGGAKQAGDV)	Rat deep vein thrombosis model	[71]
	Nattokinase-loaded and fibrinogen molecule-bound dopamine nanovesicles	Whole fibrinogen molecules	None reported	[49]
	Urokinase-loaded cRGD-bound liposomes	cRGD	Mouse mesenteric thrombosis model	[73]
	tPA-loaded cRGD-bound PEG-nanovesicle	cRGD	None reported	[69]

Fibrin-targeted	Streptokinase-bound/loaded liposome	Monoclonal antibody FnI-3C	Rat thrombosis model	[75]
	tPA-loaded fibrin-specific nanogels	Antifibrin fragment *E* polyclonal antibody	Rat disseminated intravascular coagulation model	[77]
	CREKA-modified microbubbles	CREKA	None reported	[79]
	1,1′-dioctadecyl-3,3,3′,3′-tetramethylindotricarbocyanine iodide and ticagrelor coassembly nanoparticles	CREKA	Rat carotid arterial thrombosis model	[80]

## Data Availability

No data were used to support this study.
